# Supplementary material for: mRNA-based SARS-CoV-2 vaccine candidate CVnCoV induces high levels of virus-neutralising antibodies and mediates protection in rodents
Source: NPJ Vaccines. 2021 Apr 16;6:57. doi: 10.1038/s41541-021-00311-w (PMC8052455; doi:10.1038/s41541-021-00311-w)
Supplement: Supplementary file 1 — Supplementary Information [file 41541_2021_311_MOESM1_ESM.pdf]

Supplementary Materials

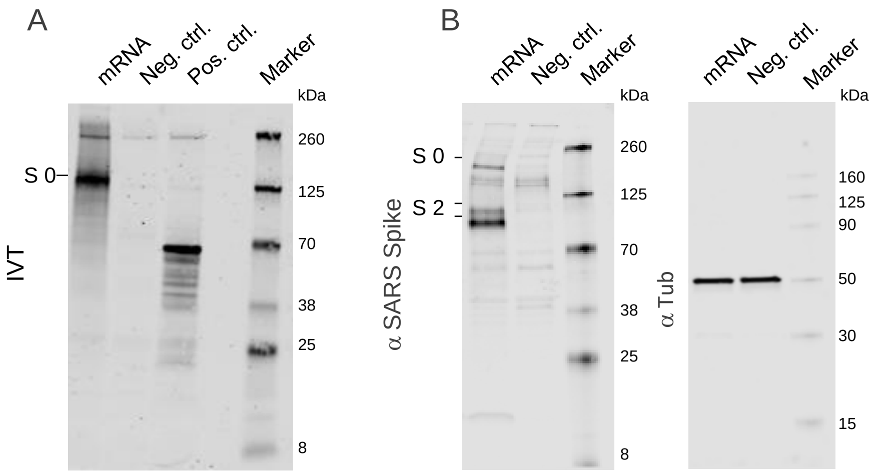

Supplementary Figure 1: Full, un-cropped images of all Western blots (A) *In vitro* translation of the mRNA component of CVnCoV in a rabbit reticulocyte lysate system. Translation of nascent proteins was detected via Western blotting. Water and luciferase control mRNA were employed as negative and positive controls, respectively. (B) HeLa cells were transfected with 2 µg of the mRNA component of CVnCoV. 24h post-transfection, cells were analysed for S expression via Western blotting using an S-specific antibody. Blots shown in B derive from the same experiment and were processed in parallel.

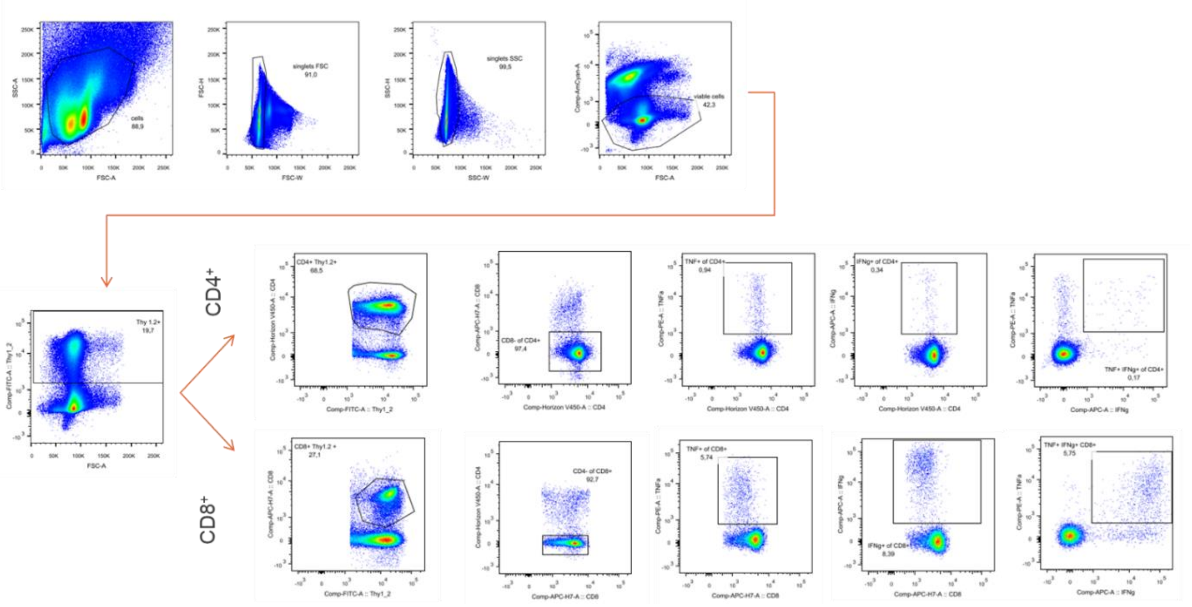

Supplementary Figure 2: Gating strategy of T cells and T cell subsets in the spleen. Female Balb/c mice were vaccinated as described. Splenocytes isolated on day 49 were stimulated using a SARS-CoV-2 spike peptide library and analysed for multifunctional IFN-γ/TNF positive T cell subsets by flow cytometry. T cells were characterized as singlets, living, Thy1.2^{+}, CD4^{+} or CD8^{+} and subdivided into the multifunctional T cell subsets based on their expression of IFN-γ and TNF.

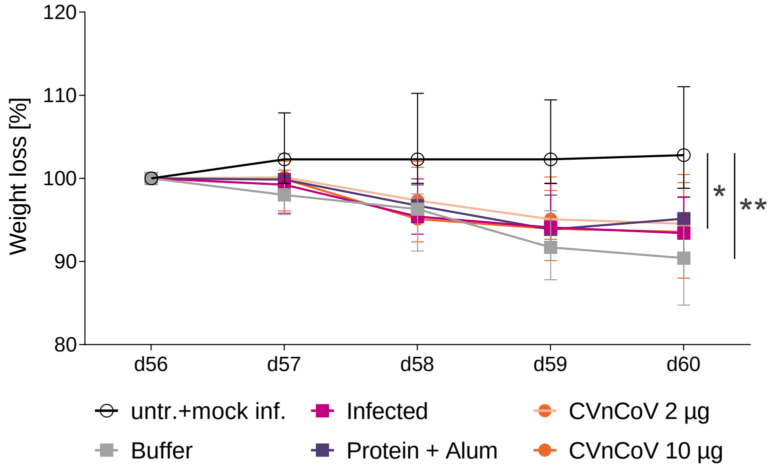

Supplementary Figure 3: Weight loss upon challenge infection. Development of weight upon challenge infection on day 56 was assessed in all groups, i.e. animals vaccinated with 10 µg or 2 µg of CVnCoV, 1.5µg of Alum adjuvanted S_{ECD} protein or buffer on day 0 and day 28, left untreated and mock infected or infected intranasally (IN) with 10^2 $TCID_{50}$/dose of SARS CoV-2 on day 0 of the experiment. Values are shown as median with range in % of value on day 56 for all groups, statistical analysis was performed using Mann-Whitney testing.

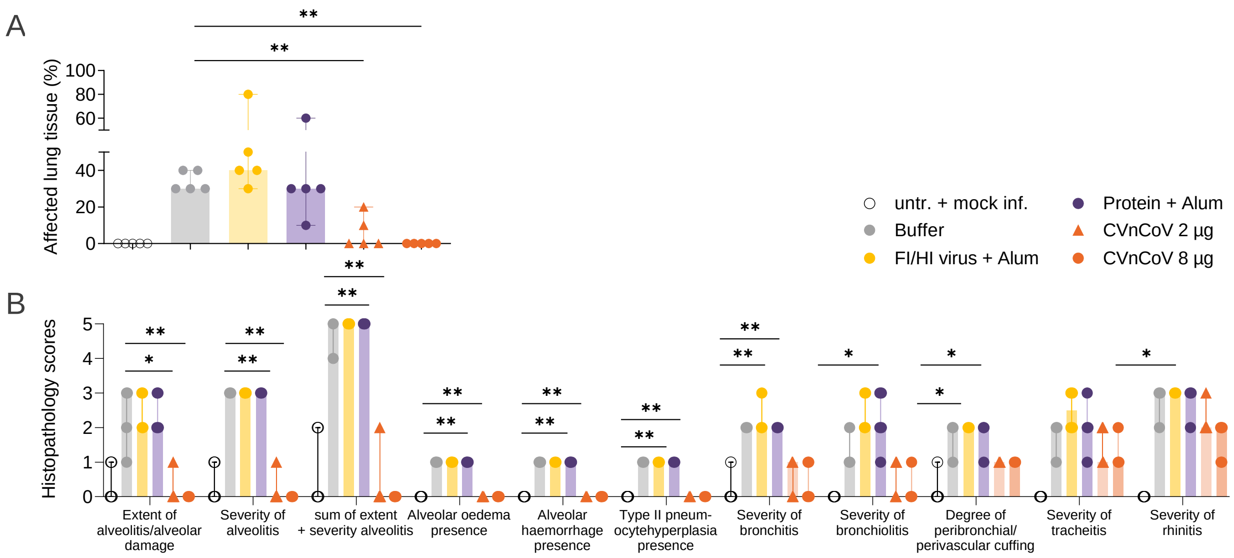

Supplementary Figure 4: Vaccination with CVnCoV protects hamsters from pathological changes of the respiratory tract on day 7 post challenge. (A) Hamsters vaccinated with 8 µg or 2 µg of CVnCoV, 1µg of Alum adjuvanted, formalin and heat inactivated SARS-CoV-2, 1.5µg of Alum adjuvanted S_{ECD} protein or buffer on day 0 and day 28, followed by of SARS CoV-2 challenge infection on d56 or left untreated and mock infected were assessed for gross pathological changes of the lungs on day 7 post challenge. Changes were determined via complete macroscopic post-mortem examination of all lung lobes. The percentage of affected lung tissue determined via visual inspection and scoring is shown. (B) Histopathological analysis was performed on day 63, seven days post challenge infection, on formalin-fixed, paraffin embedded tissues sections sampled on day 7 post challenge. Histopathological assessment scoring was performed according to severity of the inspected parameter. Each dot represents an individual animal, bars depict the median, statistical analysis was performed using Mann-Whitney testing. FI/HI virus: Formalin and heat inactivated SARS-CoV-2.
