# Supplementary material for: mRNA-based SARS-CoV-2 vaccine candidate CVnCoV induces high levels of virus-neutralising antibodies and mediates protection in rodents
Source: NPJ Vaccines. 2021 Apr 16;6:57. doi: 10.1038/s41541-021-00311-w (PMC8052455; doi:10.1038/s41541-021-00311-w)
Supplement: Supplementary file 2 — Reporting Summary [file 41541_2021_311_MOESM2_ESM.pdf]

nature research

Corresponding author(s): Susanne Rauch

Last updated by author(s): Mar 2, 2021

Reporting Summary

Nature Research wishes to improve the reproducibility of the work that we publish. This form provides structure for consistency and transparency in reporting. For further information on Nature Research policies, see our Editorial Policies and the Editorial Policy Checklist.

Statistics

For all statistical analyses, confirm that the following items are present in the figure legend, table legend, main text, or Methods section.

n/a	Confirmed	
☐	☒	The exact sample size (*n*) for each experimental group/condition, given as a discrete number and unit of measurement
☒	☐	A statement on whether measurements were taken from distinct samples or whether the same sample was measured repeatedly
☐	☒	The statistical test(s) used AND whether they are one- or two-sided<br>*Only common tests should be described solely by name; describe more complex techniques in the Methods section.*
☒	☐	A description of all covariates tested
☒	☐	A description of any assumptions or corrections, such as tests of normality and adjustment for multiple comparisons
☐	☒	A full description of the statistical parameters including central tendency (e.g. means) or other basic estimates (e.g. regression coefficient) AND variation (e.g. standard deviation) or associated estimates of uncertainty (e.g. confidence intervals)
☒	☐	For null hypothesis testing, the test statistic (e.g. *F*, *t*, *r*) with confidence intervals, effect sizes, degrees of freedom and *P* value noted<br>*Give P values as exact values whenever suitable.*
☒	☐	For Bayesian analysis, information on the choice of priors and Markov chain Monte Carlo settings
☒	☐	For hierarchical and complex designs, identification of the appropriate level for tests and full reporting of outcomes
☒	☐	Estimates of effect sizes (e.g. Cohen's *d*, Pearson's *r*), indicating how they were calculated

Our web collection on statistics for biologists contains articles on many of the points above.

Software and code

Policy information about availability of computer code

Data collection: NA

Data analysis: Graph Pad prism

For manuscripts utilizing custom algorithms or software that are central to the research but not yet described in published literature, software must be made available to editors and reviewers. We strongly encourage code deposition in a community repository (e.g. GitHub). See the Nature Research guidelines for submitting code & software for further information.

Data

Policy information about availability of data

All manuscripts must include a data availability statement. This statement should provide the following information, where applicable:

- Accession codes, unique identifiers, or web links for publicly available datasets
- A list of figures that have associated raw data
- A description of any restrictions on data availability

The authors declare that all relevant data supporting the findings of this study are available within the paper and its supplementary information files.

April 2020

Field-specific reporting

Please select the one below that is the best fit for your research. If you are not sure, read the appropriate sections before making your selection.

☒ Life sciences ☐ Behavioural & social sciences ☐ Ecological, evolutionary & environmental sciences

For a reference copy of the document with all sections, see nature.com/documents/nr-reporting-summary-flat.pdf

Life sciences study design

All studies must disclose on these points even when the disclosure is negative.

Sample size	Samples sizes in mice were determined via biometrical planning based on previously gathered data on mRNA induced SARS-CoV-2 neutralising titres
Data exclusions	NA
Replication	NA
Randomization	Animals were randomly asigned to study before the start of the experiment
Blinding	Studies were not blinded. All animal work and analysis of virus neutralising titres was performed at a CRO who had no information on specifics of the mRNA vaccines.

Reporting for specific materials, systems and methods

We require information from authors about some types of materials, experimental systems and methods used in many studies. Here, indicate whether each material, system or method listed is relevant to your study. If you are not sure if a list item applies to your research, read the appropriate section before selecting a response.

Materials & experimental systems

n/a	Involved in the study	
☐	☒	Antibodies
☐	☒	Eukaryotic cell lines
☐	☐	Palaeontology and archaeology
☐	☒	Animals and other organisms
☐	☐	Human research participants
☐	☐	Clinical data
☐	☐	Dual use research of concern

Methods

n/a	Involved in the study	
☐	☐	ChIP-seq
☐	☒	Flow cytometry
☐	☐	MRI-based neuroimaging

Antibodies

Antibodies used	Western blotting: Specific proteins were detected using rabbit anti-SARS Spike c-terminal (Abcam, Cat. ab252690) and mouse anti tubulin (Abcam, Cat. ab7291), followed by goat anti-rabbit IgG IRDye® 800CW (Li-Cor, Cat. 926-32211) and goat anti-mouse IgG IRDye® 680RD (Li-Cor, Cat. 926-68070), respectively.<br>ELISA: Mouse sera were incubated with biotin rat anti-mouse IgG1 (BD Pharmingen, Cat. 550331) or biotin rat anti-mouse IgG2a (BD Pharmingen, Cat. 550332), hamster sera with biotin goat anti-hamster (Syrian) IgG antibody (BioLegend, Cat: 405601) followed by incubation with HRP-Streptavidin (BD, Cat: 554066).<br>Flow cytometry analysis: Specific S protein expression was assessed via staining with Human anti SARS CoV S antibody (CR3022) (Creative Biolabs, Cat. MRO-1214LC) followed by goat anti-human IgG F(ab')2 fragment PE antibody (Immuno Research, Cat. 109-116-097). Cellular responses in mice were assessed using the following antibodies: anti-Thy1.2 FITC (clone 53-2.1; Biolegend, Cat.14304), anti-CD4 V450 (clone RM4-5; BD Biosciences, Cat. 560468), anti-CD8a APC-H7 (clone 53-6.7; BD Biosciences, Cat. 560182), anti-IFNy APC (clone XMG1.2, BD Biosciences, Cat. 554413) and anti-TNF PE (clone MP6-XT22, eBioscience, Cat. 25-7321-82)
Validation	NA

Eukaryotic cell lines

Policy information about cell lines

Cell line source(s)	HeLa (DSMZ, Cat. ACC 57)
Authentication	Cell lines were purchased from DSMZ and were not authenticated in house

Mycoplasma contamination	Cell lines are regularly checked for contamination by mycoplasmas
Commonly misidentified lines (See ICLAC register)	NA

Palaeontology and Archaeology

Specimen provenance	NA
Specimen deposition	NA
Dating methods	NA

☐ Tick this box to confirm that the raw and calibrated dates are available in the paper or in Supplementary Information.

Ethics oversight	*Identify the organization(s) that approved or provided guidance on the study protocol, OR state that no ethical approval or guidance was required and explain why not.*

Note that full information on the approval of the study protocol must also be provided in the manuscript.

Animals and other organisms

Policy information about studies involving animals; ARRIVE guidelines recommended for reporting animal research

Laboratory animals	Mice (female,BALB/c, 8-12 weeks of age) were obtained from Janvier Laboratories (Le Genest-Saint-Isle, France);<br>Syrian golden hamsters (female, 11 to 13 weeks old), were obtained from Envigo (Indianapolis, IN, United States).
Wild animals	NA
Field-collected samples	NA
Ethics oversight	Mice: For internal studies, experiments were approved by the Regional council Tübingen, animal testing license CUR 03/20. For studies performed externally, Balb/c mice were provided and handled by Preclinics Gesellschaft für präklinische Forschung mbH, (Potsdam, Germany). All animal experiments were conducted in accordance with German laws and guidelines for animal protection and appropriate local and national approvals<br>Hamsters: Animals were housed and all procedures were performed by Viroclinics Xplore animal facility (Schaijk, The Netherlands) under conditions that meet the standard of Dutch law for animal experimentation and in accordance with Directive 2010/63/EU of the European Parliament and of the Council of 22 September 2010 on the protection of animals used for scientific purposes. Ethical approval for the experiment was registered under protocol number AVD277002015283-WP08

Note that full information on the approval of the study protocol must also be provided in the manuscript.

Human research participants

Policy information about studies involving human research participants

Population characteristics	NA
Recruitment	NA
Ethics oversight	NA

Note that full information on the approval of the study protocol must also be provided in the manuscript.

Clinical data

Policy information about clinical studies

All manuscripts should comply with the ICMJE guidelines for publication of clinical research and a completed CONSORT checklist must be included with all submissions.

Clinical trial registration	NA
Study protocol	NA
Data collection	NA
Outcomes	NA

Dual use research of concern

Policy information about dual use research of concern

Hazards

Could the accidental, deliberate or reckless misuse of agents or technologies generated in the work, or the application of information presented in the manuscript, pose a threat to:

No	Yes	
☒	☐	Public health
☒	☐	National security
☒	☐	Crops and/or livestock
☒	☐	Ecosystems
☒	☐	Any other significant area

Experiments of concern

Does the work involve any of these experiments of concern:

No	Yes	
☒	☐	Demonstrate how to render a vaccine ineffective
☒	☐	Confer resistance to therapeutically useful antibiotics or antiviral agents
☒	☐	Enhance the virulence of a pathogen or render a nonpathogen virulent
☒	☐	Increase transmissibility of a pathogen
☒	☐	Alter the host range of a pathogen
☒	☐	Enable evasion of diagnostic/detection modalities
☒	☐	Enable the weaponization of a biological agent or toxin
☒	☐	Any other potentially harmful combination of experiments and agents

ChIP-seq

Data deposition

☐ Confirm that both raw and final processed data have been deposited in a public database such as GEO.

☐ Confirm that you have deposited or provided access to graph files (e.g. BED files) for the called peaks.

Data access links<br>*May remain private before publication.*	NA
Files in database submission	NA
Genome browser session<br>(e.g. UCSC)	NA

Methodology

Replicates	NA
Sequencing depth	NA
Antibodies	NA
Peak calling parameters	NA
Data quality	NA
Software	NA

Flow Cytometry

Plots

Confirm that:

☒ The axis labels state the marker and fluorochrome used (e.g. CD4-FITC).

☒ The axis scales are clearly visible. Include numbers along axes only for bottom left plot of group (a 'group' is an analysis of identical markers).

☒ All plots are contour plots with outliers or pseudocolor plots.

☒ A numerical value for number of cells or percentage (with statistics) is provided.

Methodology

Sample preparation	For intracellular cytokine staining (ICS), splenocytes from vaccinated and control mice were isolated and single cell suspensions were prepared in supplemented medium. 2x10^6 splenocytes (200 µl) per well were stimulated for 5-6 h at 37 °C using an SARS-CoV-2 peptide library (JPT, PM-WCPV-S2) at 0.5 µg/ml. After 1 h Golgi Plug (BD Biosciences, Cat: 555029) was added in a dilution of 1:200 (50 µl) to the splenocytes to inhibit the secretion of intracellular cytokines. After stimulation, splenocytes were centrifuged, resuspended in supplemented medium and stored at 4 °C overnight. Following this, splenocytes were washed twice in PBS and stained with AquaDye (Invitrogen, Cat: L34957) solution at 4 °C for 30 min. After an additional washing step in FACS buffer (PBS with 0.5% BSA) cells were surface stained for Thy1.2, CD4 and CD8 and incubated with FcyR-block for 30 min at 4 °C in FACS buffer. After surface staining, splenocytes were washed in FACS buffer and fixed using Cytofix/Cytoperm (BD Biosciences) according to the manufacturer's instructions. After fixation, splenocytes were washed in perm buffer and stained for IFN-γ and TNF for 30 min at 4 °C. After staining, the cells were washed with perm buffer, resuspended in FACS buffer supplemented with 2mM EDTA and 0.01% Natriumacid and stored at 4 °C until analysis. The following antibodies were used for flow cytometry analysis: anti-Thy1.2 FITC (clone 53-2.1; Biolegend, Cat.14304), anti-CD4 V450 (clone RM4-5; BD Biosciences, Cat. 560468), anti-CD8a APC-H7 (clone 53-6.7; BD Biosciences, Cat. 560182), anti-IFNγ APC (clone XMG1.2, BD Biosciences, Cat. 554413) and anti-TNF PE (clone MP6-XT22, eBioscience, Cat. 25-7321-82).
Instrument	Splenocytes were analysed on a Canto II flow cytometer (BD Biosciences)
Software	Flow cytometry data were analyzed using FlowJo software (Tree Star, Inc, Ashland, USA.).
Cell population abundance	NA
Gating strategy	T cells were characterized as singlets, living, Thy1.2+, CD4+ or CD8+ and subdivided into the multifunctional T cell subsets based on their expression of IFN-γ and TNF.

☒ Tick this box to confirm that a figure exemplifying the gating strategy is provided in the Supplementary Information.

Magnetic resonance imaging

Experimental design

Design type	NA
Design specifications	NA
Behavioral performance measures	NA

Acquisition

Imaging type(s)	NA
Field strength	NA
Sequence & imaging parameters	NA
Area of acquisition	NA

Diffusion MRI ☐ Used ☐ Not used

Preprocessing

Preprocessing software	NA
Normalization	NA
Normalization template	NA

Noise and artifact removal	NA
Volume censoring	NA

Statistical modeling & inference

Model type and settings	NA
Effect(s) tested	NA

Specify type of analysis: ☐ Whole brain ☐ ROI-based ☐ Both

Statistic type for inference (See Eklund et al. 2016)	*Specify voxel-wise or cluster-wise and report all relevant parameters for cluster-wise methods.*
Correction	*Describe the type of correction and how it is obtained for multiple comparisons (e.g. FWE, FDR, permutation or Monte Carlo).*

Models & analysis

n/a	Involved in the study
☐	☐ Functional and/or effective connectivity
☐	☐ Graph analysis
☐	☐ Multivariate modeling or predictive analysis

Functional and/or effective connectivity	NA
Graph analysis	NA
Multivariate modeling and predictive analysis	NA
